# Determinants of anxiety and depression and their association with coping strategies in health professionals in war and conflict-afflicted areas

**DOI:** 10.3389/fpsyt.2026.1728734

**Published:** 2026-02-25

**Authors:** Maisa Nabulsi, Muna Ahmead, Nuha El Sharif

**Affiliations:** 1 Faculty of Pharmacy, Al-Quds University, Jerusalem, Palestine; 2 Faculty of Public Health, Al-Quds University, Jerusalem, Palestine

**Keywords:** anxiety, coping strategies, depression, health professionals, health workers, Palestine

## Abstract

**Introduction:**

little is known about the impact of conflict or war on Palestinian mental health professionals, as well as their strategies for dealing with these problems. Given the continuous war in Palestine, the purpose of this study was to investigate the prevalence of depression, anxiety, and coping strategies among healthcare professionals.

**Methods:**

The study utilized a cross-sectional research design. Self-reported questionnaires included the Hospital Anxiety and Depression Scale (HADS) and the Brief COPE scale was used. The relationships between the study’s variables were examined using descriptive statistics (frequencies and percentages), Pearson correlation, and multivariate regression.

**Results:**

A total of 713 health professionals were recruited. Anxiety and depression had a prevalence of 51.5% and 45.3%, respectively. The multivariate analysis revealed that those who utilized self-distraction, active coping, substance use, emotional support, and acceptance as coping strategies were less likely to suffer depression. Also, participants who employed denial, venting, and planning as coping strategies were more likely to develop depression. In addition, males were less likely to experience anxiety than females, and those who utilized emotional support and acceptance as coping mechanisms were less likely to feel anxious. However, participants who utilized denial, behavioral disengagement, venting, and planning were more likely to experience anxiety.

**Conclusion:**

The findings revealed a high prevalence of depression and anxiety among health professionals during wartime and political violence. As a result, health professionals need assistance in enhancing their mental well-being through psychological support, psychotherapy, and comprehensive training in stressor management.

## 1 Introduction

Healthcare workers face unique challenges, including inadequate funding, high patient volumes, and trauma from conflict, which can lead to emotional distress and burnout (1). Additionally, healthcare workers around the world confront numerous work-related challenges that can have a significant impact on their mental health. These include heavy workloads, long shifts, fast-paced environments, a lack of proper safety measures, the continuous nature of patient care, ethical dilemmas, worries about job security, violence at work, and a lack of social support (2). Consequently, healthcare providers face conflicts in their work life due to the inherent tension between their duty of care and the demanding nature of healthcare environments (3). Burnout, depression, anxiety, sleeplessness, and other mental health problems might result from this psychological distress (4, 5). Anxiety and depression are rather common, according to research (7, 8). Anxiety affects 33–50% of healthcare professionals (6), and depression affects 26–60% (8, 9). Mental disorders profoundly affect both individuals and healthcare organizations. Previous research indicates that chronic illnesses, such as cardiovascular disease, along with physical symptoms like lethargy, dizziness, vomiting, nausea, disturbed sleep, and muscular spasms, may be caused by mental disorders (10). Also, healthcare workers who suffer from mental illnesses are more likely to have negative attitudes toward their jobs, poor judgment, and low performance (11), in addition to being more likely to quit, be absent, or have high turnover rates.

An increase in stress levels may have an effect on healthcare workers’ capacity to cope (5, 12, 13). According to Folkman et al., coping involves how people think and behave in response to stressful situations (14). Folkman and Lazarus identified two main types of coping strategies: problem-focused coping, which aims to change the current situation or find a solution to the problem, and emotion-focused coping, which deals with the emotional distress that comes with stressful situations (15). Two types of coping mechanisms are linked to improved mental health outcomes and are regarded as adaptive (16–18). Avoidant coping strategies—including drug abuse, self-distraction, behavioral disengagement, and denial—are considered maladaptive or dysfunctional (16). Since 1967, in Palestine, the West Bank and Gaza Strip have been under Israeli occupation. The Palestinian Ministry of Health (MOH), United Nations Relief and Works Agency for Palestine Refugees in the Near East (UNRWA), non-governmental organizations (NGOs), and the private sector oversee primary, secondary, and tertiary healthcare in Palestine (19). According to the Palestinian Ministry of Health (2017), there are 81 hospitals under their authority. The West Bank (including East Jerusalem) hosts 51 of these hospitals, while Gaza hosts 30 (20). After October 7, 2023, the health status in Palestine was significantly impacted, and the healthcare system in Palestine continues to confront serious challenges as a result of conflict, political instability, and underfunding (12). Depression and post-traumatic stress disorder are common among healthcare workers since they are the first to see and treat serious injuries caused by military operations (21). Additionally, healthcare personnel encounter unique challenges such as working in an unsafe environment, insufficient resources, an excessive number of patients, and conflict-related trauma, which can result in burnout, anxiety, and depression (5, 12). Assessing the magnitude and underlying causes of depressive and anxiety symptoms in healthcare workers, along with their coping strategies, may provide a basis for formulating and executing intervention strategies for policymakers and healthcare organizations to mitigate, manage, and alleviate the impact of these symptoms. However, there has been little study on the mental health of healthcare personnel during conflict, specifically the association between depression, anxiety, and coping strategies. Therefore, the aim of this study was to assess the prevalence of depression and anxiety symptoms and to examine their associations with sociodemographic characteristics and coping strategies among healthcare workers. Additionally, it sought to determine the factors that affect the prevalence of depression and anxiety symptoms among these healthcare professionals.

## 2 Materials and methods

### 2.1 Participants and procedure

The research was a descriptive cross-sectional survey conducted from January 15, 2024, to February 1, 2024, following two months after the beginning of the Gaza War. We previously published the initial part of the study, which examined the association between burnout and coping strategies in healthcare professionals (5). The current study is a continuation of prior research that focuses on coping strategies, anxiety, and depression among the same sample of health professionals, as described elsewhere (5). It targeted all Palestinian health professionals presently employed in Palestine during the continuing Gaza conflict and political conflict, including doctors, nurses, pharmacists, and allied professions (e.g., anesthetic technicians, radiologic technicians, and medical laboratory personnel). Participants were selected by convenience and snowball sampling techniques. Data were collected using an anonymous online self-administered questionnaire. In light of Israeli military restrictions on mobility and closures in the West Bank and Jerusalem, participants were asked to complete an online questionnaire created with Google Forms. Participants received the study link via various channels, including social media, WhatsApp, emails, and organizational websites. Additionally, participants were requested to disseminate the link among mental health professionals nationwide, resulting in 713 responses from Jerusalem and the West Bank.

### 2.2 Data collection instruments

The study used a self-reported questionnaire, which had the following three sections:

Section one included a socio-demographic sheet designed to collect information about the participants’ age, gender, place of residence, marital status, occupation, workplace, education level, monthly income, and years of experience. It also specified the governorates: the northern governorate included Nablus, Jenin, Qalqilia, Tulkarim, Tubas, and Salfeet; the middle governorate included Ramallah, East Jerusalem, and Jericho; and the southern governorate included Hebron and Bethlehem.

The second section had the Hospital Anxiety and Depression Scale (HADS), which is a 14-item scale created to assess the presence of anxiety and depression. The HADS creates two scales to distinguish the two states: HADS–A for anxiety (seven questions) and HADS–D for depression (seven questions). On a 4-point severity scale, items are rated, and each question is scored between 0 (no impairment) and 3 (severe impairment), with three denoting the highest anxiety or depression level. A case is considered conclusive if the score on either scale is greater than or equal to 11. A score of 0–7 indicates normal, 8–10 indicates mild anxiety/depression, 11–14 indicates moderate anxiety/depression, and a score of 15–21 is equal to severe anxiety/depression. The internal consistency coefficient (Cronbach’s α) was 0.831.

The third section had the Brief COPE scale, which consisted of 28 questions. Both cognitive and behavioral strategies of coping are included, and for each category, respondents indicate whether they have used a coping response on a four-point Likert scale (1 = I have not been doing this at all; 2 = I have been doing this a little bit; 3 = I have been doing this an average amount; 4 = I have been doing this a lot), and the higher score represents greater coping strategies used by the respondents. The Brief COPE scale assesses the following coping mechanisms: self-distraction, active coping, denial, substance use, emotional support, instrumental support, behavioral disengagement, venting, positive reframing, planning, humor, acceptance, religion, and self-blame. The internal consistency coefficient (Cronbach’s α) was.850.

The survey was translated into Arabic and back into English. The Arabic terminology was piloted by 20 health professionals and reviewed by five experts to verify accuracy and understandability.

### 2.3 Statistical analysis

The data were analyzed using SPSS version 25 (IBM Corp., Chicago, Illinois, USA). A descriptive analysis was performed using the frequencies and percentages for the categorical variables. The chi-squared test was used to analyze the relationships between depression, anxiety, the sociodemographic variables, and coping strategies. Statistically significant variables were further analyzed with multivariate logistic regression. A p-value < 0.05 was considered sufficient for statistical significance. Adjusted odds ratio and 95% confidence interval were also reported.

### 2.4 Ethical approval and consent to participate

The Declaration of Helsinki was followed in the implementation of all study methods. Al Quds University Research Ethical Committee approval was obtained (Ref No: 347/REC/2023). There was anonymity in this online survey. At the outset of the survey, written information was given regarding its goal and the intended use of the data. By completing the questionnaire, the participants gave their informed consent to take part in the research.

## 3 Results

### 3.1 Description of the sample

The sample included 713 healthcare workers, of which 60.3% were female. 53.2% were between the ages of 18 and 30, 43% were single, and 47.3% earned above $1150 monthly. Nursing made up 57.2% of the participants, pharmacists 8.4%, and physicians 15.6%. Furthermore, 70% possessed a bachelor’s degree, and 46.1% had been employed in their current job for over 6 years as seen in **Table 1**.

**Table 1:** Sociodemographic characteristics, occupation, and work conditions of study participants.

Characteristics		Frequency (N)	Percent (%)
Gender	Male	283	39.7%
	Female	430	60.3%
Age (years)	18-30	379	53.2%
	31- 40	191	26.8%
	41+	143	20.1%
Living place	City	341	47.8%
	Village	331	46.4%
	Refugee camp	41	5.8%
Marital Status	Single	308	43.2%
	Not single*	405	56.8%
Monthly Income (US$)	No income	50	7.0%
	< 570	52	7.3%
	570-1150	275	38.6%
	1151 -1700	209	29.3%
	1701+	127	17.8%
Occupation	Allied profession	134	18.8%
	Physicians	111	15.6%
	Pharmacists	60	8.4%
	Nurses	408	57.2%
Work Place	Governmental	461	64.7%
	Private	172	24.1%
	Others**	80	11.2%
Governorate	North	230	32.3%
	top	221	31.0%
	South	262	36.7%
Education level	Bachelor	504	70.7%
	Higher studies (Master and Ph.D degrees)	209	29.3 %
Years of experience	< 1	147	20.6%
	1-3	120	16.8%
	4-6	117	16.4%
	7-10	90	12.6%
	11-15	112	15.7%
	16+	127	17.8%

*Married/widowed/divorced **Non-governmental organizations, civil institutes, and international agencies.

### 3.2 Prevalence of depression and anxiety

**Figure 1:**
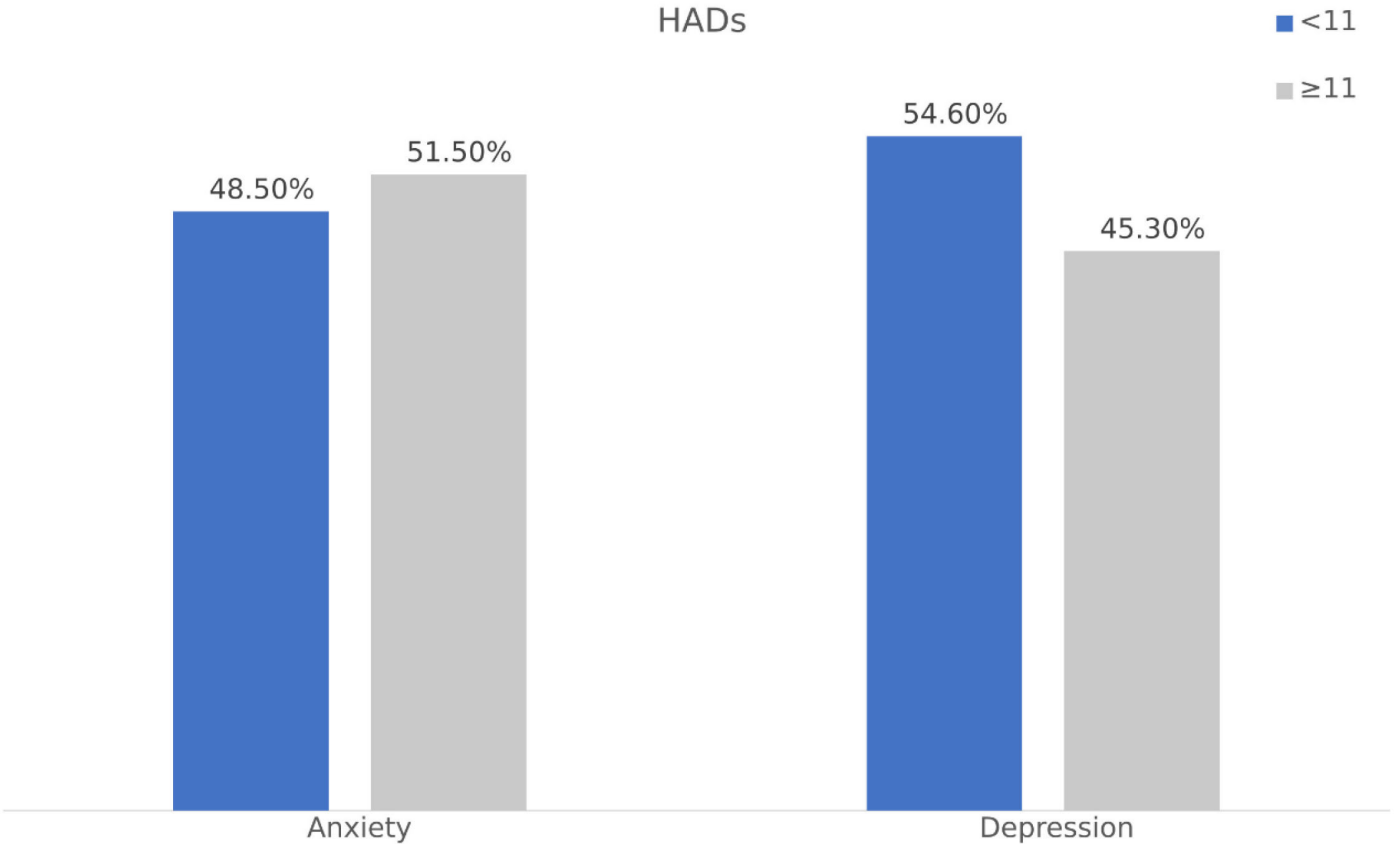
The prevalence of depression and anxiety symptoms.

The results showed that 45.3% of the sample met the clinical threshold for depression (a score of 11 or above), indicating an increased likelihood of obtaining a clinical diagnosis. Furthermore, 51.5% of the sample reached the clinical threshold for anxiety, as seen in **Figure 1**.

### 3.3 Association between depression, anxiety, and sociodemographic variables

Pearson’s chi-square was performed to assess the associations between depression symptoms and respondent characteristics. The results, as seen in **Table 2**, demonstrated significant relationships between depression and age (p<.033) and governorate (p<.015). Significant correlations were also found between anxiety and gender (p <.007) and monthly income (p <.024).

**Table 2:** The association between depression, anxiety and sociodemographic variables of study sample.

Characteristics		Depression					Anxiety				
		<11		≥11		P value	< 11		≥11		P value
		F	%	F	%		F	%	F	%	
Gender	Male	150	38.6%	133	41.2%	.478	155	44.8%	128	34.9%	.007*
	Female	239	61.4%	190	58.8%		191	55.2%	239	65.1%	
Age	18 - 30 years	219	56.3%	160	49.5%	.033*	176	50.9%	203	55.3%	.319
	>30- 40 years	89	22.9%	102	31.6%		93	26.9%	98	26.7%	
	>40- years	81	20.8%	61	18.9%		77	22.3%	66	18.0%	
Occupation	Allied profession	69	17.7%	65	20.1%	.683	60	17.3%	74	20.2%	.503
	Medicine	61	15.7%	50	15.5%		59	17.1%	52	14.2%	
	Pharmacist	30	7.7%	30	9.3%		32	9.2%	28	7.6%	
	Nursing	229	58.9%	178	55.1%		195	56.4%	213	58.0%	
Work place	Government	261	67.1%	199	61.6%	.309	230	66.5%	231	62.9%	.544
	Private	88	22.6%	84	26.0%		81	23.4%	91	24.8%	
	Others	40	10.3%	40	12.4%		35	10.1%	45	12.3%	
Place of residency	City	192	49.4%	149	46.1%	.389	179	51.7%	162	44.1%	.111
	Village	172	44.2%	158	48.9%		147	42.5%	184	50.1%	
	Refugee camp	25	6.4%	16	5.0%		20	5.8%	21	5.7%	
Marital status	Single	170	43.7%	138	42.7%	.793	140	40.5%	168	45.8%	.152
	Not single	219	56.3%	185	57.3%		206	59.5%	199	54.2%	
Monthly Income (US $)	No income	29	7.5%	21	6.5%	.511	19	5.5%	31	8.4%	.024*
	< 570	23	5.9%	29	9.0%		18	5.2%	34	9.3%	
	570-1150	148	38.0%	127	39.3%		127	36.7%	148	40.3%	
	1151 -1700	115	29.6%	93	28.8%		112	32.4%	97	26.4%	
	1701+	74	19.0%	53	16.4%		70	20.2%	57	15.5%	
Governorate	North	124	31.9%	105	32.5%	.015*	117	33.8%	113	30.8%	.512
	top	137	35.2%	84	26.0%		109	31.5%	112	30.5%	
	South	128	32.9%	134	41.5%		120	34.7%	142	38.7%	
Education level	Bachelor degree	271	69.7%	233	72.1%	.470	243	70.2%	261	71.1%	.795
	Higher studies	118	30.3%	90	27.9%		103	29.8%	106	28.9%	
Years of experience	> 1 year	78	20.1%	69	21.4%	.114	58	16.8%	89	24.3%	.236
	1-3 years	70	18.0%	50	15.5%		61	17.6%	59	16.1%	
	4-6 years	70	18.0%	47	14.6%		56	16.2%	61	16.6%	
	7-10 years	40	10.3%	50	15.5%		48	13.9%	42	11.4%	
	11-15 years	55	14.1%	57	17.6%		57	16.5%	55	15.0%	
	16+	76	19.5%	50	15.5%		66	19.1%	61	16.6%	

*The Chi-square statistic is significant at the.05 level.

### 3.4 Associations between coping strategies, depression and anxiety

Pearson correlation was used to investigate the associations between coping strategies and depression symptoms and anxiety, as seen in **Table 3**. For anxiety, there were significant positive correlations between anxiety and self-distraction, denial, use of instrumental support, behavioral disengagement, planning, venting, humor, and self-blame, and a negative correlation with acceptance. Furthermore, there were significant negative correlations between depression and self-distraction, active coping, and acceptance. Moreover, there was a positive correlation between depression and denial, venting, and planning.

**Table 3:** Associations between coping strategies and depression and anxiety.

Coping strategy	Anxiety		Depression	
	Pearson correlation	P value	Pearson correlation	P value
Self-distraction	.088^*^	.019	-.142^**^	.000
Active coping	-.026	.486	-.123^**^	.000
Denial	.241^**^	<.001	.135^**^	.000
Substance use	.028	.457	-.054	.151
Use of emotional support	.048	.198	-.072	.055
Use of instrumental support	.102^**^	.007	-.049	.188
Behavioral disengagement	.215^**^	<.001	.046	.221
Positive reframing	.008	.831	-.003	.943
Venting	.208^**^	<.001	.109^**^	.004
Planning	.201^**^	<.001	.122^**^	.001
Humor	.093^*^	.013	-.050	.180
Acceptance	-.123^**^	.001	-.092^*^	.014
Religion	.023	.532	.019	.620
Self-blame	.212^**^	<.001	.094^*^	.012

^∗^p < 0.05, ** p < 0.05.

### 3.5 Multivariate logistic regression for determinants of depression and anxiety

Multivariate logistic regression was used to explore the factors that predict the development of depression symptoms (**Table 4**). The findings showed that the participants who used self-distraction (AOR:.785, p <.001), active coping (AOR:.860, p <.012), substance use (AOR:.851, p <.030), emotional support (AOR:.877, p <.020), and acceptance as coping strategies (AOR:.893, p <.045) were less likely to experience depression than those who did not use them. However, the participants who used denial (AOR: 1.153, p <.006), venting (AOR: 1.254, p <.001), and planning (AOR: 1.290, p <.001) as coping strategies were more likely to experience depression than those who did not use them. Moreover, multivariate logistic regression was used to explore the factors that predict the development of anxiety symptoms (**Table 4**). The findings showed that males were less likely to experience anxiety than females (AOR:.616, p <.004). Also, the participants who used emotional support (AOR:.867, p <.010) and acceptance as coping strategies (AOR:.788, p <.000) were less likely to experience anxiety than those who did not use them. However, the participants who used denial (AOR: 1.149, p <.007), behavioral disengagement (AOR: 1.155, p <.022), venting (AOR: 1.214, p <.004), and planning (AOR: 1.308, p <.000) were more likely to experience anxiety than those who did not use them.

**Table 4:** Multivariate logistic regression models for the predictions of depression and anxiety symptoms.

Characteristics	Sig.	AOR	95% CI	
			Lower	Upper
Depression				
Self-distraction	.001	.785	.694	.888
Active coping	.012	.860	.765	.968
Denial	.006	1.153	1.042	1.276
Substance use	.030	.851	.736	.984
Use of emotional support	.020	.877	.784	.980
Venting	.001	1.254	1.099	1.431
Planning	.001	1.290	1.138	1.462
Acceptance	.045	.893	.799	.997
Anxiety				
Gender ( Ref. female)	.004	.616	.443	.857
Denial	.007	1.149	1.038	1.272
Use of emotional support	.010	.867	.778	.967
Behavioral disengagement	.022	1.155	1.021	1.306
Venting	.004	1.214	1.064	1.385
Planning	.000	1.308	1.146	1.492
Acceptance	.000	.788	.705	.880

The multivariate logistic regression model includes all study variables.

AOR, Adjusted Odds Ratio; 95% CI, 95% Confidence Interval; Sig., Significance level (p <0.05).

## 4 Discussion

The current study is a pioneer in investigating the prevalence of depression and anxiety, as well as their association with coping strategies, among Palestinian health workers in a wartime context. Our findings underscore the importance of taking mental health during conflict periods seriously as a public health concern. The findings revealed a significant prevalence of depression and anxiety among Palestinian health workers. These results are considered high in comparison to the findings of previous studies. Researchers reported that the prevalence of depression among health professionals in Uganda was 11.0%, while the prevalence of anxiety was 14.5% (22). According to Elhadi et al., 45% of emergency doctors in Libya had anxiety and depression following the civil war (23). One systematic review found that the prevalence of anxiety among healthcare professionals was 23.2%, while depression was 22.8% (24). In Ukraine, Rzońca et al. reported that anxiety and depression disorders were prevalent among physicians and paramedics during combat at 12.6% and 9%, respectively (25).

Furthermore, Lim et al. revealed that the prevalence of depression and anxiety increased during times of conflict or war by 28.9, and 30.7, respectively (26). Additionally, during the COVID-19 epidemic in the United States, Prasad et al. found that 22.7% of healthcare professionals were depressed and 69.4% were anxious (27).

Nevertheless, other studies revealed a higher prevalence than our study. For instance, a recent study in Gaza found that 93.9% of health professionals experienced depression and 96.5% experienced anxiety (21). Additionally, Wańkowicz et al. found that depression and anxiety were prevalent among healthcare workers in Poland during the COVID-19 pandemic, with 70.7% and 64.3%, respectively (28). The findings of our study may be attributed to the fact that Palestinian healthcare workers were working in a resource-limited environment, where they were confronted with significant stressors such as inadequate staffing, limited health support, and heavy responsibilities (29). In addition, practitioners’ mental health problems may be exacerbated by the ubiquitous hardships associated with armed conflicts, including poverty, malnutrition, devastation, economic and social deterioration, and pervasive feelings of sadness, fear, despair, and depression (30). To effectively manage stress and prioritize their own mental well-being, it is imperative that health personnel receive psychological interventions and treatment, particularly in high-stress contexts such as wars.

Several variables have been identified as potential causes of anxiety and depression among mental health professionals during armed conflict and hostilities in the current study. For instance, the results indicated that males were less likely to experience anxiety than females, which is consistent with the findings of other studies (24, 31). Research indicates that women exhibit a significantly higher prevalence of anxiety disorders and a higher level of anxiety sensitivity than men (32). Furthermore, women display greater emotional distress and anxious coping strategies in response to stressors than their male counterparts (32, 33). Additionally, women adopt a ruminative coping style (34). Research indicates that an increasing trajectory of anxiety arousal symptoms in women is caused by a negative cognitive style or rumination in conjunction with a stressor (35, 36). This trend was attributed by Zhang et al. to the fact that females are required to work night schedules and have contact with high-risk patients, which may exacerbate their symptoms of anxiety (37). Consequently, to address the needs of female health professionals, psychological intervention and psychotherapy are required in the workplace. Furthermore, additional research is required to investigate the cause of the elevated anxiety levels among female health workers.

The present study revealed that mental health professionals employed varied coping strategies, which might decrease or increase their levels of depression and anxiety. For instance, the current study found that mental health professionals who used self-distraction, substance use and active coping were less susceptible to depression than their counterparts who did not. This finding is consistent with the results of other studies (30, 38–40) and contradicts other studies (41). Sharif et al. reported comparable findings, indicating that participants utilized adaptive coping strategies, including substance use, self-distraction, and active coping, to manage their depression (42). Also, Wahab et al. revealed that depression was less likely to occur in correlation with active coping and self-distraction strategies (43). Further, Avants et al. found that depressed patients were more likely to rely on avoidant coping strategies and less on adaptive coping strategies (44). For instance, distraction is a passive coping strategy that involves the individual avoiding confrontation with the situation or attempting to resolve the problem (45). If the situation is unchangeable, it may be beneficial to engage in activities that provide a sense of relaxation, such as reading, exercising, or viewing television, to divert one’s attention from the distressing event (45). Furthermore, individuals with depression may be particularly susceptible to engaging in substance use as a coping mechanism to mitigate their severe, all-encompassing psychological distress (39). Research has linked efficient coping strategies, such as problem-solving, to improved mental health outcomes. However, maladaptive strategies, such as substance misuse or avoidance, can exacerbate depression (46). According to Steare et al., the persistent use of avoidant coping strategies can reduce quality of life by inducing ongoing stress (47). Additionally, individuals who use substances to alleviate negative emotions may be at increased risk of developing substance use disorders (48, 49). Our findings underscore the potential benefits of addressing depression, which is a promising target for interventions that foster adaptive coping and empowerment among health professionals who are depressed. In addition, it is important to provide psychotherapies specifically tailored to address substance use. Furthermore, additional research is required to evaluate the potential efficacy of these strategies in reducing problematic substance use among individuals with depression.

Additionally, our results indicated that the participants who employed emotional coping strategies, including acceptance and emotional support, were less likely to experience anxiety and depression, which is consistent with the findings of other studies (50–52). In contrast, Yoon et al. found that depression was negatively associated with problem-focused coping, while emotion-focused coping was positively associated with it (53, 54). Emotional support is crucial because it is regarded as a protective mechanism against life stressors and is believed to enhance health and wellness (55). Furthermore, acceptance serves as an effective psychological buffering mechanism (41). According to Stals et al., acceptance may serve as a means of coping with the bereavement process when confronted with overwhelming illness, trauma, and loss in the context of restricted resources (41). Our results may indicate that depressed participants exhibited a tendency to reduce their use of the “confrontation” coping strategy and were more likely to rely on acceptance to manage their stressors, which may have contributed to their depression and anxiety. Nevertheless, Beck et al. suggested that acceptance may exacerbate pessimistic emotions (56). Consequently, it was essential to implement coping skills training and psychological therapies in the care of professionals with depression and anxiety (56). The coping intervention has to include the reconstruction of recognition and the promotion of a positive view regarding adaptation to stress.

Finally, our research indicated that participants employing avoidant coping mechanisms (denial), emotional coping strategies (venting), and problem-solving strategies (planning) were more vulnerable to experiencing depression and anxiety than those who did not use these methods, which confirms findings from other studies (30). Avoidant coping mechanisms are frequently considered maladaptive because of their poor correlation with psychological well-being (57). Wahab et al. found that the use of maladaptive coping techniques, such as denial, correlated with a heightened probability of depression (43). In addition, Lee et al. showed that elevated utilization of emotion-focused coping strategies correlates with increased depression symptoms, particularly in relation to emotional venting and denial coping (58). Excessive venting without effective coping methods may result in rumination, reinforcing negative thought patterns and intensifying depression symptoms (59). Furthermore, denial coping often involves the suppression or disregard of negative feelings, especially those associated with depression (58). According to Bjørkløf et al., health workers risk missing out on opportunities for processing emotions, acknowledging them, and getting the help they need if they avoid or reject these feelings (60). In addition, our finding indicated that planning may exacerbate anxiety and depression. This finding stands in contrast to previous studies (50–52) that have shown planning coping as a protective factor against anxiety and depression. A potential explanation is that the presence of an excessive number of complicated and unpredictable factors in a healthcare environment can impede the planning process and, in fact, increase tension (41). Lazarus and Folkman’s transactional theory posits that the effectiveness of any coping strategy depends fundamentally on the controllability of the stressor (61). Problem-focused coping strategies, such as planning, are most effective when stressors are manageable and can be directly addressed. Nevertheless, when stressors are outside an individual’s control, problem-focused strategies may prove counterproductive (15). They may lead individuals to allocate cognitive and emotional resources to unresolvable issues, resulting in heightened exhaustion and anxiety (62). Moreover, wartime conditions impose significant, often uncontrollable pressures on healthcare professionals. Factors such as ongoing violence, unpredictable attacks, lack of resources, damage to infrastructure, and threats to personal safety are out of their control (63). Thus, sticking rigidly to a planning strategy in chaotic situations can hinder emotional adaptation, which is crucial for achieving psychological resilience during wartime. Therefore, relying on planning under these circumstances may demonstrate cognitive inflexibility and an inability to adjust coping strategies to meet situational demands, thereby suggesting a maladaptive coping approach (64). As a consequence, healthcare providers can express their emotions and investigate alternative approaches by providing education about healthy coping strategies, including the limitations of excessive venting or denial coping (65). Also, psychoeducation promotes the cultivation of healthier coping strategies and builds awareness (58). Zhang and Li emphasized the significance of offering psychological interventions such as community-based programs, internet- and mobile-mediated interventions, cognitive behavioral therapy, mindfulness meditation, trauma-informed care, and support groups for individuals affected by armed conflict (66).

This research is subject to limitations. The representativeness of the sample and the generalizability of the findings are influenced by convenience sampling and cross-sectional designs, which reduce the ability to demonstrate causal relationships and limit the generalizability of results. Additionally, the use of a self-reported questionnaire may increase the likelihood of reporting bias. It is likely that health personnel who are already employed in the impacted areas, including the Gaza Strip, do not have access to or the opportunity to use this technology, as recruiting took place using platforms such as Google Docs and WhatsApp. Consequently, the representativeness of the sample may be affected by this circumstance. In addition, there is a scarcity of research that investigates the coping strategies, anxiety, and depression of healthcare professionals during wartime. Consequently, the ability to compare our results to those of other studies is restricted. Despite these limitations, the results of the current study shed light on the psychological well-being of health professionals residing in countries that are conflict-affected. This study is a significant contribution to the current literature, as it is the first to examine the coping strategies, depression, and anxiety of Palestinian healthcare professionals during periods of political violence and armed conflict.

### 4.1 Implications for clinical care and policy

The high prevalence of depression and anxiety among healthcare personnel points to the urgent need for specialized mental health interventions and support in the healthcare sector and public action to protect the mental health of these professionals. Promoting early detection and proactive intervention for depression and anxiety is essential as a preventive strategy against severe mental health disorders. Regular mental health assessments, counseling services, and educational initiatives bolster the resilience and coping abilities of health professionals, particularly among females and individuals employing avoidance strategies such as denial and substance use, as well as emotional coping techniques like venting and problem-solving strategies such as planning. Consequently, educating individuals on healthy coping strategies and offering psychoeducation fosters awareness and the cultivation of adaptive coping mechanisms. Moreover, collaboration among governments, healthcare institutions, and communities is essential to acknowledge and bolster the mental health of healthcare personnel. Additionally, further research is necessary to investigate the reasons behind the elevated prevalence of depression and anxiety, particularly among female health workers. Additional research is necessary to assess the potential efficacy of coping strategies in mitigating problematic substance use among healthcare professionals suffering from depression and anxiety.

## 5 Conclusion

The findings revealed a high prevalence of depression and anxiety among health professionals during wartime and political violence. As a result, health professionals need assistance in enhancing their mental well-being through psychological support, psychotherapy, and comprehensive training in stress management.

## Data Availability

The original contributions presented in the study are included in the article/supplementary material. Further inquiries can be directed to the corresponding author.
